# Antiretroviral Therapy in HTLV-1 Infection: An Updated Overview

**DOI:** 10.3390/pathogens9050342

**Published:** 2020-05-01

**Authors:** Francesca Marino-Merlo, Emanuela Balestrieri, Claudia Matteucci, Antonio Mastino, Sandro Grelli, Beatrice Macchi

**Affiliations:** 1 IRCCS Centro Neurolesi Bonino-Pulejo, 98123 Messina, Italy; fmarino@unime.it; 2 Department of Experimental Medicine, University of Rome “Tor Vergata”, 00133 Rome, Italy; balestrieri@med.uniroma2.it (E.B.); matteucci@med.uniroma2.it (C.M.); grelli@med.uniroma2.it (S.G.); 3 Department of Chemical, Biological, Pharmaceutical, and Environmental Sciences, University of Messina, 98166 Messina, Italy; 4 The Institute of Translational Pharmacology, Consiglio Nazionale delle Ricerche (CNR), 00133 Rome, Italy; 5 Department of Chemical Science and Technologies, University of Rome “Tor Vergata”, 00133 Rome, Italy

**Keywords:** HTLV-1, antiretrovirals, HAM/TSP, ATL

## Abstract

The human T cell leukemic/lymphotropic virus type 1 (HTLV-1), discovered several years ago, is the causative agent for a rapid progressive haematological malignancy, adult T cell leukemia (ATL), for debilitating neurological diseases and for a number of inflammatory based diseases. Although the heterogeneous features of the diseases caused by HTLV-1, a common topic concerning related therapeutic treatments relies on the use of antiretrovirals. This review will compare the different approaches and opinions in this matter, giving a concise overview of preclinical as well as clinical studies covering all the aspects of antiretrovirals in HTLV-1 infection. Studies will be grouped on the basis of the class of antiretroviral, putting together both pre-clinical and clinical results and generally following a chronological order. Analysis of the existing literature highlights that a number of preclinical studies clearly demonstrate that different classes of antiretrovirals, already utilized as anti-HIV agents, are actually capable to efficiently contrast HTLV-1 infection. Nevertheless, the results of most of the clinical studies are generally discouraging on the same point. In conclusion, the design of new antiretrovirals more specifically focused on HTLV-1 targets, and/or the establishment of early treatments with antiretrovirals could hopefully change the perspectives of diseases caused by HTLV-1.

## 1. Introduction

The human T cell leukemic/lymphotropic virus type 1 (HTLV-1) [1] was the first human retrovirus to be identified, almost 40 years ago [2]. It has been estimated, some years ago, that at least 5–10 million people were infected with HTLV-1 worldwide [3]. Data on the prevalence of HTLV-1 infection mainly derive from studies carried out in ancient endemic areas such as Japan, Carribean islands, some regions in South America, sub-Saharian Africa, and the Middle East. More recently, Australia, where about 50% of Australian indigenous were recently reported to be HTLV-1 infected [4], has also been added to the list of these ancient endemic areas. However, mainly due to increased immigration and tourist fluxes in the recent years, we can reasonably suppose that the virus is not yet confined in ancient endemic areas and the current prevalence of HTLV-1 infection in the world population is actually unknown.

HTLV-1 is the etiological agent of a rapid progressive malignancy, Adult T cell leukemia (ATL), which develops in 5% of infected people, as well as a debilitating neurological disease, HTLV-1 associated myelopathy/tropical spastic paraparesis (HAM/TSP), which develops in 4% of HTLV-1 positive individuals. In addition, a number of inflammatory HTLV-1 associated diseases such as uveitis, Hashimoto’s thyroiditis and Graves’ disease, HTLV-1 associated pulmonary disease, infective dermatitis associated with HTLV-1, HTLV-1 associated inflammatory myositis, and HTLV-1 associated arthritis have been reported [5]. HTLV-1 is usually transmitted through sexual partners, breastfeeding, blood transfusion, and recently, it was reported also through organ transplantation. Although HTLV-1 was the first human retrovirus discovered before the most recent HIV-1, both diagnosis and therapeutic approach of infection await further clarification and investigations. There are a number of peculiar aspects of HTLV-1 infection which clearly distinguish it from HIV-1 infection and allow the virus to escape host control by causing a latent infection. HTLV-1 is a highly cell associated virus which mainly integrates influencing through its regulatory protein the function of host cells. Differently from HIV-1, HTLV-1 virus spread relies mainly on vertical transmission (mitotic spread), while a role can be also played by horizontal transmission (infectious spread). In contrast with HIV-1 which kills target CD4+ cells, HTLV-1 infection is characterized by expansions and persistence of infected T cell clones that prompt the emergence of both neurological and haematological diseases. Mechanisms regulating the clonality of HTLV-1 infected cells have not been fully elucidated [6]. Although diseases induced by HTLV-1 infection are characterized by highly heterogeneous symptoms and clinical manifestation, one of the most and for a long time debated topic concerning the management of HTLV-1 infected patients regards, in general, the use of antiretrovirals. In fact, antiretroviral agents are though to act by specifically targeting viral enzymes involved in viral replication. In particular, according to accumulated experience for HIV infection, the main potential targets for antiretrovirals are RNA reverse transcription, DNA integration, and viral polyprotein cleavage. Consequently, antiretrovirals might be active only on productively infected cells, i.e., towards the infectious spread, but not on non-producing infected cells, i.e., towards the mitotic spread. Since, in HTLV-1 infected patients, usually very low (if any) productive infection is detected, antiretrovirals are expected to be of low efficacy, unless viral replication is activated. This could easily explain the low efficacy of antiretrovirals in HTLV-1 infection compared with HIV infection. Thus, given that the opportunity to treat HTLV-1 infected individuals with antiretroviral therapy is still controversial, in this review, we will try to compare the different approaches and opinions concerning this matter. To this purpose, we will give a concise, and as complete as possible, overview of preclinical as well as clinical studies covering all the aspect of antiretrovirals in HTLV-1 infection. Studies, after a brief note on the early experimental approaches, have been grouped by class of antiretrovirals and, where suitable, disease. Obtained results will be presented mainly in a chronological order, deliberately putting together preclinical and clinical research. 

## 2. Pioneering Studies

Zidovudine (3’-azido-3’-deoxythymidine; AZT), a nucleoside analogue acting as competitive inhibitor of reverse transcriptase was the first effective antiretroviral used in HIV infection [7]. The potential effect of AZT against HTLV-1 was firstly shown by an in vitro study using a CD4+ cell clone, established from primary lymphocytes following co-culture with a lethally irradiated HTLV-1 producing tumour cell line. This study highlighted a profound suppression of GAG production and proviral DNA in the presence of AZT [8]. Soon after, the effects of AZT towards HTLV-1 infection in vivo were demonstrated by Isono et al. in an animal model of ATL in rabbits. They hypothesized a duplex role of AZT that in rabbit could interfere with leukaemogenesis by both inhibiting reverse transcriptase, and by decreasing the growth of inoculated transformed leukemic cells [9]. Thus, the era of antiretrovirals in HTLV-1 infection was opened.

## 3. Translational Approaches with Nucleoside/Nucleotide Reverse Transcriptase Inhibitors in HTLV-1 Infection: Preclinical and Clinical Studies

### 3.1. Neurological Diseases

An HTLV-1 associated neuromyelopathy, originally named tropical spastic paraparesis (TSP), was first uncovered in the Caribbean area. A similar neurological disorder was then described in Japan and named as HTLV-1 associated myelopathy (HAM). The two diseases were then recognized to be identical and here we refer to this neurological disease, occurring in HTLV-1 infected patients, as HAM/TSP. First clinical trials using AZT in HTLV-1-infected HAM/TSP patients provided conflicting reports. An open study conducted for six months on five patients (1 g/day) affected by HAM/TSP observed no effect of AZT on the encephalomyelopathy and therefore no clinical benefit [10]. Conversely, an open label study with ten HAM/TSP patients, scored for expanded disability status scale (EDSS) treated with high dose of AZT (2 g/d for four weeks, followed by 1 g/d for 20 weeks) reported an objective improvement in seven patients and no general worsening [11]. Some years later, studies by our group, aimed at controlling in vitro viral transmission and its inhibition by AZT, clearly confirmed that AZT, when added in culture at time zero of infection with HTLV-I, had a marked protective effect on proviral DNA, RNA, and GAG expression even at the lowest concentration of 100 nM [12]. On the other hand, in the same study, as expected, no evidence of antiretroviral effects by AZT on PBMC already infected by coculture with MT-2 was observed.

Obviously, since HAM/TSP is caused by HTLV-1 infection, the assessment of the effect of antiretroviral therapy on viral load could highlight the possible influence of inhibition of reverse transcription on the clinical response. However, in vivo response to AZT was variable, and therefore, studies with other nucleoside/nucleotide reverse transcriptase inhibitors (NRTIs) were performed. In particular, it was reported in observational studies that treatment with lamivudine (2,3-dideoxy-3-thiacytidine; 3TC) for 24 weeks was able to reduce HTLV-1 viral load in five out of five patients with HAM/TSP, but with oscillation rising toward the baseline, accompanied by parallel variation in CTL precursor frequency and CD25 expression. These data pointed out an effect of therapy towards reverse transcription and the return to base line proviral DNA was interpreted as a failure in drug phosphorylation within the cell or emergence of viral variants with less susceptibility to 3TC inhibition [13]. Moreover, it was excluded that the return to base line proviral DNA level could be owed to mutations since after extensive analysis of the consensus sequence of RT before and after 3TC treatment no substantial substitutions were found. This was an important proof of concepts, although variation in the response of HAM/TSP patients was not easily understood. Resistance of HTLV-1 to 3TC was further highlighted by in vitro assays. Five different HTLV-1 isolates were found to be susceptible to a number of NRTIs, AZT, didanosine (2′,3′-dideoxyinosine; ddI), stavudine (2,3-didehydro-3 –deoxythymidine; d4T), and zalcitabine (2,3-dideoxycytidine; ddC), but not to 3TC. Resistance of HTLV-1 RT to 3TC was acknowledged in HIV and ascribed to the conserved LPQG (Q151M) or YMDD (M184) mutations in HIV-RT, but the portion of alignment of HIV RT and HTLV-1 RT corresponding to 3TC resistance did not match excluding that resistance to 3TC treatment of HTLV-1 was owed to this type of mutation [14]. Soon after, our group demonstrated that 3TC exerted the capability to inhibit HTLV-1 infection of peripheral blood mononuclear cells (PBMC) in culture, by suppressing viral RNA and protein expression, but only at very high IC50 of 100 µM, i.e., with antiviral potency 100 times lower in comparison with previous data obtained with AZT [15]. On the basis of the transient decline of HTLV-1 viremia in response to therapy with 3TC in HTLV-1 infected patients, it was hypothesized that partial resistance of HTLV-1 to 3TC was of natural type, due to polymorphism at codon 118 (valin replaced by isoleucin). This was further supported by evidence that isoleucine at position 118 induced resistance to 3TC incorporation in HIV RT which shares a similar sequence with the HTLV-1 RT [16]. Therefore, the presence of isoleucine at the position 118 could be responsible of natural resistance of HTLV-1 to 3TC, although these data have not been confirmed by biochemical and site-directed mutagenesis studies. Nevertheless, these results justified a warning regarding the NRTIs therapy, including the use of 3TC, in HTLV-1 patients. In vitro assays on the effects towards HTLV-1 infection, of several NRTIs including AZT, 3TC, d4T, ddC, abacavir ((1S,4R)-4-[2-amino-6-(cyclopropylamino)purin-9-yl]cyclopent-2-en-1-yl] methanol, ABC), and the acyclic analog of deoxyadenosine 5′-monophosphate nucleotide tenofovir (9-(R)-[2- (phosphonomethoxy)propyl] adenine, PMPA; TFV) were improved by studies using recombinant HTLV-I vectors to reproduce early steps of the viral replication. These studies analyzed the effects of antiretrovirals on HTLV-1 infection in vitro using the sophisticated tool of single-cycle infection and reached conclusions similar to those achieved through classical methods of multiple-cycle infection through HTLV-1 transmission in vitro by co-cultivation [17]. Further, our group assayed TFV, showing encouraging results in vitro consisting of high protection of PBMC infection by HTLV-1 after pre-treatment with 1 µM and direct inhibition of HTLV-1 reverse transcriptase activity in a cell-free assay [18]. Importantly, the same study demonstrated that TFV was endowed with a selectivity index four times higher than that of AZT. Clinical studies on the use of NRTIs in HAM/TSP remained open, small and uncontrolled until 2006, when a randomized, double-blind, placebo controlled study was carried out in sixteen patients treated with a combination of AZT and 3TC for 48 weeks. The results showed that the combined treatment was unable to inhibit proviral load as well as to improve the clinical state of patients. Several hypotheses for lacking of effect by AZT+3TC combination in hampering HTLV-1 proviral load could be formulated. Considering that successive intracellular phosphorylation of nucleoside analogues to their triphosphate forms is required for the pharmacological activity of these compounds, one possibility is that a lack of activity can be due to an inadequate concentration of intracellular phosphorylated antiretrovirals needed to inhibit elongation of DNA chain. On the other hand, lack of efficacy could be due to the limited role played by HTLV-1 reverse transcription in the spread of virus infection in vivo at the stage of infection when treatment was initiated. Actually, as previously discussed, HTLV-1 is spread horizontally from cell-to-cell as well as vertically through cell division in HAM/TSP patients, and therefore it is unclear which of the two could be prevalent in blown HTLV-1 related neurological diseases [19]. Anyhow, authors predicted for more detailed studies since the therapy was not toxic. In fact, a later study was aimed to investigate the response to NRTIs ex vivo by directly assessing the HTLV-1 RT activity in samples from patients. The RT activity of cultured PBMC from six patients under 3TC+AZT combination therapy and/or under tenofovir disoproxil fumarate (TDF) monotherapy was assayed at time 0 an after 48 months of treatment. Although RT preparations from samples of patients were always sensitive to the in vitro inhibitory effect of NRTIs, except for 3TC, no decrease in RT activity was observed after 48 months of in vivo therapy in samples from patients. Therefore, the trend of HTLV-1 RT activity in samples from treated patients simply paralleled that of proviral load, which was subjected to oscillation but was basically very similar to the baseline [20]. In addition, also in those cases in which pol deletion was detected, samples from patients retained sensitivity to NRTIs in the in vitro RT assay, suggesting that the lack of virological response was not owed to outcome of mutant strains during therapy. Taken together, these data suggest that combination of AZT+3TC according to the treatment schedule utilized in the above mentioned study, did not exert effects both on viral load and on RT activity in HTLV-1 infected neurological patients. One general conclusion of the above reported study, contributing to keep the attention on NRTIs, was that they should be preferentially active for pre- or post-exposure prophylaxis rather than in therapy in HAM/TSP. Again, also in this case failure might depend on the fact that treatment of HAM/TSP with NRTIs could require a longer time, or to the fact that the time when treatment was initiated in the cohort of patients was too late. Interestingly, a more recent case report sustained an impressive clinical improvement of the neurological state of a HAM/TSP patient following a prolonged AZT+3TC treatment at an advanced stage of disease, leaving still open the way for NRTIs in neurological disorders caused by HTLV-1 [21]. In this case, however, there was no report on viral load. One aspect that must be highlighted concerning all the above mentioned preclinical and clinical studies is that they utilized only NRTIs previously developed as anti-HIV compounds. Potentially, on the other hand, the design and the development of new, anti-HTLV-1 specific NRTIs could be extremely useful. The first studies in vitro on the effect of NRTIs towards HTLV-1 infection using compound never assayed before against HIV, were performed in our laboratory. In fact, a new class of NRTIs, named phosphonated carbocyclic 2′-oxa-3′-aza-nucleosides, showed an HTLV-1 RT inhibitory activity in vitro at the concentration of about 1 nM, i.e., similar to that exerted by TVF and AZT [22,23]. They showed activity ex-vivo also towards RT from HAM/TSP patients [20], but they were not further developed for in vivo studies. Thus, in vitro studies repeatedly demonstrated the suitability of using NRTIs to inhibit HTLV-1 infection but, unfortunately, they were not enough potent in in vivo therapy in HAM/TSP, when used according to the protocols set up in the above mentioned studies and the clinical stage of the enrolled patients, except for one reported case.

One alternative strategy for the use of NRTIs in HAM/TSP could be a combination treatment including compounds acting on different targets from viral replication. Such a possible alternative approach was the investigation of the effects of a combination treatment of NRTIs with the epigenetic regulator valproic acid (VPA). Asymptomatic baboon naturally infected with STLV-1, almost identical to HTLV-1 in humans, were treated with a combination of AZT plus VPA resulting in a reduction of proviral load by 5–12-fold in 50% of the animal tested at early stage of infection. The treatment with VPA was shown to be associated to a transient rise in the proviral load early in the first week of treatment while combination with AZT prevented the rebound of the viral load. Given that the decrease of viral load in VPA/AZT treatment was accompanied by an increase of CD8+ effector-cell cytotoxic activity, it was hypothesized that the decrease in viral load was owed to the reactivation of the immune response against the virus facilitated by AZT-mediated inhibition of viral transmission. On the other hand, the rebound of viral load following interruption of the combined treatment might be due to virus production by reservoirs not affected by the therapy, similar to what is observed in HIV infection. On the basis of the results obtained in STLV-1 infection, the combination treatment with VPA and AZT could be potentially transferred to HAM/TSP patients if treated at an early stage of the disease [24].

Taken together, studies carried out until now on NRTIs in HAM/TSP provide conflicting results, but it seems that there is no opinion excluding their potential effect at least at some stage of the disease. Accordingly, attention has been recently paid to a novel generation of NRTIs anti-HIV drugs, i.e., the acyclic nucleoside phosphonates (ANP). In particular, prodrugs of the already mentioned TFV, also named as PMPA [9-R-(2-phosphonomethoxypropyl)adenine], were investigated in a new model of HTLV-1 infection in vitro using as recipient cells LTR luciferase reporter Jurkat T-cells [25]. Interestingly the results showed that the RT inhibitors, acyclic nucleotide analogue of adenosine adefovir (or PMEA), and its pro drug, adefovir dipivoxil (or bis-POM-PMEA) were found to be more active than AZT in inhibiting HTLV-1 infection in vitro while the pro-drugs of PMPA, such as tenofovir disoproxil (bis-POC-PMPA), TDF and TAF (tenofovir alafenamide) had more effect than TFV. These in vitro results encouraged the use of TDF in combination with valproate acid in STLV-1 naturally infected primates, as preclinical models of asymptomatic carriers of HTLV-1.

Thus, clinical studies concerning antiretrovirals in HTLV-1 induced neurological disorders provide, at the moment, insufficient evidence to support the use of antiretroviral therapy for the treatment of HAM/TSP as asserted in a recent systematic review and consensus-based recommendations [26]. Nevertheless, this does not exclude the need of further investigation on this issue.

### 3.2. Haematological Diseases

In contrast to HAM/TSP, NRTIs were fruitfully used to treat ATL caused by HTLV-1 infection. This choice was due to the poor therapeutic options for treating ATL which was resistant to the classical cytotoxic chemotherapy. One of the first reports about the use of antiretrovirals was done by Gill et al. in 1995 when AZT was combined with interferon alpha (IFNa) [27]. This combination caused 28% of complete remission and in general a major response in 58% of the 19 treated individuals, with overall median survival of three months. However, these results were rather similar to those obtained following treatment with chemotherapy. Therefore, no conclusion on the superiority of AZT plus IFNa over classical chemotherapy could be drawn. Similar results were obtained in AZT plus IFNa treatment of five ATL patients, one with smoldering and four with acute ATL, of whom three resulted complete responders while two partial responders [28]. The longest survival was 27 months after the diagnosis. One key, unanswered question was whether the combination AZT+IFNa was efficacious owed to its antiviral effect or whether acted as cytotoxic drugs. To clarify this aspect, assumptions and further studies have been made. Among the first issues investigated were the effects of AZT plus IFNa on cell proliferation, on the expression of apoptosis regulators proteins, on cell growth, and on the cell cycle [29]. Results of this study, performed using HTLV-1 chronically infected cell lines as well as PBMC from ATL patients, apparently excluded a direct cytotoxic effect of AZT plus IFNa in ATL. Interestingly, in vitro studies by our group demonstrated that AZT by itself induces cell death very poorly in uninfected as well as HTLV-1 infected cells, but its cytotoxic activity could be greatly enhanced by a concomitant repression of the anti-apoptotic response through inhibition of NF-kB activation [30,31]. Nevertheless, the effect of AZT+IFNa in ATL might be more complex involving activation of antiviral immunity and/or other variants in vivo. Although mechanisms of the combination therapy with AZT+IFNa in ATL were not disclosed, successive studies further confirmed the efficacy of this treatment. A longer follow up of 15 adult T cell leukemia/lymphoma, ATL patients treated over four years with the combination of AZT plus IFNa, resulted in 18 months median survival time with no major side effects [32]. Again, events involved in the improvement of patients treated with the combination treatment were not clarified; however, it was hypothesized that an increase of the CTL response against the virus could be involved. Moreover, considering the response exerted by the combination treatment versus that of the classical chemotherapy, and that a preventive chemotherapeutic treatment able to reduce the tumour burden, proved to enforce the response to AZT+IFNa, furthers studies on the effects of a combination chemotherapy followed by AZT+IFNa in ATL were planned. The effect of AZT+IFNa combination treatment in ATL patients was later confirmed in a study carried out in a different country that demonstrated inhibition of viral load and decrease of angiogenesis. Unfortunately, the number of followed patients in this study was limited, with a total of nine patients treated only for three months and therefore it was difficult to draw a conclusion in the long run [33]. Interestingly, the efficacy of AZT+IFNa was overall proved by a meta-analysis performed on 254 patients with ATL. Actually, in acute ATL, achievement of complete remission by antiviral therapy caused 82% five-year survival, while in chronic and smoldering ATL, antiviral therapy resulted in 100% five-year survival [34]. Moreover, a retrospective study on the outcome of AZT+IFNa treatment in 73 patients with aggressive ATL recruited in England from 1999–2009, was reported. This study extended the treatment strategy and showed that AZT+IFNa as “combined first line therapy” or as “deferred therapy” when used after relapse, were both associated to a prolonged overall survival in comparison with chemotherapy alone [35]. However, this retrospective study suffered from the heterogeneity of the patients in terms of previous and later treatments, the later including transplantation, or times of initiation of AZT+IFNa treatment. Actually, a new prospective that arose from this study was that AZT+IFNa could be a maintenance therapy for those patients not eligible for transplantation. Successive studies characterized the molecular response of primary ATL tumours from patients treated with AZT+IFNa and showed that resistance to antiviral treatment was associated with the upregulation of a subunit of the NF-kb systems, c-Rel, and to the upregulation of interferon regulatory factor-4. The study hypothesized that these factors could predict the response to therapy of ATL patients and that the AZT+IFNa combination was a suppressive therapy rather than a curative one. This implies that ATL patients should remain on therapy for a long term [36]. Collectively, these studies demonstrated that AZT treatment in combination with IFNa was clearly associated with the best achievable clinical response, even if variable in length and, definitely, subjected to relapse. 

### 3.3. Haematological Diseases: Direct or Indirect Effect of AZT+IFNa?

The above reported studies highlighted the need to elucidate whether AZT+IFNa combination exerted a direct antiretroviral effect. Actually, an indirect antiretroviral effect by AZT+IFNa combination was recently hypothesized as the consequence of an impact towards dendritic cells or macrophages in which viral replication occurs and that provide growth factors and cytokines useful for the survival of ATL cells [37]. Interestingly, recent data have supported a role for IFNa in suppressing in vitro intracellular HTLV-1 Tax protein expression in IL-2 dependent HTLV-1 infected T cells, mediated by upregulation of an RNA-dependent protein kinase (PKR) [38]. In addition, IFNa alone upregulated the NF-kB system in IL-2 dependent HTLV-1 infected T cells in vitro. In this study, the role of AZT in combination with IFNa was unclear, since it was shown that the NRTI cooperated with IFNa in inducing apoptosis by upregulating p53 expression, but no direct evidence for induction of regulated cell death by AZT+IFNa treatment in HTLV-1 immortalized cells was observed. Thus, even in this study, the role of AZT as a direct anti-HTLV1 agent still remained elusive [38]. All together, these data suggest that a univocal response for explaining the outcome of AZT+IFNa combination treatment in vivo cannot be simply deduced from in vitro studies. However, differences in results of various in vitro and in vivo studies might be due to different experimental procedures, such as modalities of infection, uptake by different target cells, technical differences in read-out of the assays. Nevertheless, the direct targeting of HTLV-1 reverse transcription by the NRTI plus IFNa treatment in vivo could not be excluded. In fact, by means of ex vivo assays, therapy with AZT and IFNa has been recently demonstrated to completely inhibit RT activity and to affect the clonality pattern in samples from responding ATL patients. These data showed that a decrease of RT activity in the blood was predictive of response to combination therapy in ATL patients, suggesting that HTLV-1 RT could be directly targeted by AZT+IFNa treatment in vivo [39]. A recent case report described a long-term remission, six years after end of therapy with AZT+IFNa, in an ATL patient. The residual disease was demonstrated through a high throughput proviral integration site mapping, which showed a five years lasting delayed reduction in the amount of the malignant clone. This slow response suggested that the AZT+IFNa therapy could not have exerted any direct cytotoxic or antiviral effect towards infected cells [40]. Considering the substantial lack of information regarding the optimal length for the combination treatment with AZT+IFNa in ATL and the insufficiency of biomarkers for response to therapy, a sensitive method to detect residual disease could be useful to clarify the long term response to AZT+IFNa therapy. Moreover, clinical studies drew attention to a triple combination in which arsenic trioxide was added to AZT+IFNa. In particular, the efficacy and safety of this triple combination was first reported in a pilot study on newly diagnosed chronic ATL [41], and, more recently, a retrospective clinical analysis has shown that arsenic trioxide consolidation in combination with low-dose AZT+IFNa maintenance enhanced long-term disease control in a small cohort of ATL patients [42]. In conclusion, the issue of whether the beneficial effect of AZT+IFNa in ATL could be due to a direct antiviral action or to indirect mechanisms is worthy of further investigation. Nevertheless, ATL researchers and clinicians concluded in their consensus meeting report of the 18th International Conference on Human Retrovirology/Human T-Lymphotropic Virus and Related Retroviruses that the extinction of viral replication by AZT could hopefully contribute, in combination with treatments targeting the transforming activity of HTLV-1, to theoretically eradicate the disease [43].

### 3.4. Transplantation

Transplantation has been recently recognized as a potentially efficacious HTLV-1 transmission route. The lack of routinely screening of organ donors for HTLV-1 makes difficult to precisely state that the transplant is actually responsible for ATL or HAM/TSP development in the recipient. Some scattered case reports were reported in the literature regarding HTLV-1 transmission through organ transplantation in France, Spain, and United States [44,45,46,47]. Antiretroviral therapy based on AZT and the integrase inhibitor raltegravir (for this compound, see also the next paragraph) was recommended as postexposure prophylaxis within 48 h from transplant before establishing of infection through generation of proviral DNA [48]. Actually, the screening of organ donors was introduced in 2012 in UK after a case of transmission of HTLV-1 from a single solid organ donor to three transplant recipients. A liver and two kidneys were taken from a deceased woman, and their HTLV-1 positivity was established early after transplant. The recipients underwent an early treatment with AZT and raltegravir for 24–54 days and at 30 months after the treatment no outcome of HTLV-1 associated diseases was observed. However, molecular and serological studies revealed the presence of HTLV-1, 16 days after transplantation [49]. Antiretroviral treatment was initiated at day 23, i.e., when infection was already established and, therefore, presumably without impact on already infected cells. In fact, antiretroviral treatment did not arrest rapid dissemination of HTLV-1, with a proviral load plateau at six weeks and unique integration site analysis indicating early clonal expansion and high rate of infectious spread. This lays for the need of using antiretroviral as prophylactic treatment in case of pre transplantation assessment of HTLV-1 positivity of the donor or at very early time after transplantation in case of post-transplantation assessment. Nevertheless, precious data of this study do not exclude that antiretrovirals could have a role in limiting HTLV-1 spread in transplanted patients. Recently, the development of HAM/TSP was described in a case of kidney transplant from a cadaveric donor which was found HTLV-1 positive 24 h after transplantation. The recipient underwent antiretroviral prophylaxis with AZT, 3TC, and raltegravir that was maintained only for one month. The patient did not seroconvert for a month and virus was not present until day 83 from transplant. However, three months after transplantation the patient was found positive for HTLV-1 provirus. Antiretroviral therapy was resumed and maintained for six months, but neurological symptoms progressed even in presence of steroid treatment [50]. Presumably, antiretroviral treatment delayed virus appearance but successive virus spread was not controlled due to early interruption of therapy. Another case report from Spain accounted for a woman who received renal transplant from HTLV-1 positive donor and started antiretroviral therapy with AZT, 3TC and raltegravir for 18 months. Eight months after transplantation, the patient developed HAM/TSP showing high proviral load. The second kidney transplant recipient from the same donor was treated with antiretroviral therapy for two months after transplantation. A low viral load was found, the patient underwent transplant rejection, but he did not develop any disease associated to HTLV-1 infection after three years. Altogether, these reports indicate that antiretroviral treatment started after transplantation, and even at ab early time, cannot overcome HTLV-1 infection transferred with the transplant, but rather delay infection depending also on the length of treatment. Since the reported cases of transplant recipient from HTLV-1 positive donors subjected to antiretroviral therapy including NRTIs are few, and there remains a lack of established protocol for antiretroviral treatment, it is still difficult to draw definitive conclusions concerning this subject.

## 4. Translational Approaches with Integrase and Protease Inhibitors in HTLV-1 Infection

Beside NRTIs, only two other classes of antiretroviral agents were translated from HIV experience to possible anti-HTLV-1 drugs. They belong to the classes of integrase and protease inhibitors. No study on anti-HIV non-nucleoside reverse transcriptase inhibitors in HTLV-1 infection has been reported.

Several integrase strand transfer inhibitors (INSTIs) have been developed and its use has been introduced in HIV treatment [51]. Unfortunately, preclinical and clinical studies on the INSTIs activity towards HTLV-1 infection are still limited. Early studies demonstrated for the first time that HIV integrase inhibitors, namely diketo acids and styrylquinolines, were able to inhibit strand-transfer reaction carried on by HTLV-1 encoded integrase in a cell free model, by directly inhibiting the number of integration events [52]. Further studies showed that the above mentioned INSTIs variably inhibited proviral load spread in PBMC experimentally infected in vitro with HTLV-1 through co-cultures. Successive studies demonstrated that other anti-HIV INSTIs, specifically raltegravir and isentress, inhibited HTLV-1 integrase in an in vitro cell-free infection with recombinant virus vectors showing an IC50 between 1 and 25 nM. Also in this case, the inhibition of HTLV-1 infection was demonstrated to depend on a decrease of HTLV-1 integration, as assessed through the Alu assay for viral integration [53]. Unfortunately, however, monotherapy with raltegravir in five HTLV-1 infected patients, two of whom with HAM/TSP, showed only a transient inhibition of viral load after six months of therapy which could not be maintained in the following six months. The blip of the virus was not owed to the outcome of drug resistance during treatment. This recalls the above reported cases of HAM/TSP patients under treatment with NRTIs whose viral load was inhibited only through the first six months of therapy [19]. Nevertheless, hopefully, perspective will be derived from in vitro studies on more recent INSTIs. Actually, the recent FDA approved anti-HIV INSTIs bictegravir was shown to inhibit HTLV-1 integration in a cell-free assay and HTLV-1 infection in a cell-to-cell transmission assay, three and five times, respectively, more than raltegravir. It is still unclear, however, why bictegravir was about 20 times less efficient in inhibiting HTLV-1 integration than HTLV-1 infection in vitro. It was hypothesized that this discrepancy might be dependent on the high efficiency of the drug to enter into the target cells [54]. Collectively, these data suggest that INSTIs inhibited HTLV-1 integration and infection in vitro while the inhibition of proviral load by raltegravir in patients was transient. Therefore, the use of INSTIs and RT inhibitors in HTLV-1 poses the same unanswered question, i.e., whether their use is more suitable as preventative/prophylaxis approach in acute phase of infection or to preventively inhibit mother to born transmission rather than as a late therapeutic approach in patients suffering from full-blown diseases years after primary infection. 

Regarding protease inhibitors, only one recent evidence for the effect of an already used anti-HIV agent belonging to this class has been reported in [55]. Ritonavir was shown to inhibit NF-kB activation in Tax transfected lymphoid cell line, and inhibition of NF-kB activation regulated apoptotic genes. The effect was further proven in an animal model of ATL in NOG mice. Following the injection of ATL cells, NOG mice developed weight loss, and cachexia, in addition to the enlargement of lymph nodes, spleens, lungs. Treatment with ritonavir decreased lymphocytes infiltration and organ infiltration into lymph nodes, spleen, lungs, and liver. These results sustain that ritonavir exerted an antitumoral effect likely by inhibiting NF-kB activation. Although the evidence of a potential effect of protease inhibitors in HTLV-1 infection is still limited, the hypothesized mechanism of action on NF-kB system activation encourage further detailed studies. Actually, one common and crucial feature of HTLV-1 infected/transformed cells is the up regulation of NF-kB very likely induced by transactivation of Tax protein [56]. Thus, the role of protease inhibitors in HTLV-1 infection could be targeting cellular determinants induced by infection rather than directly affecting the assembly of viral particles.

## 5. Conclusions

**Figure 1:**
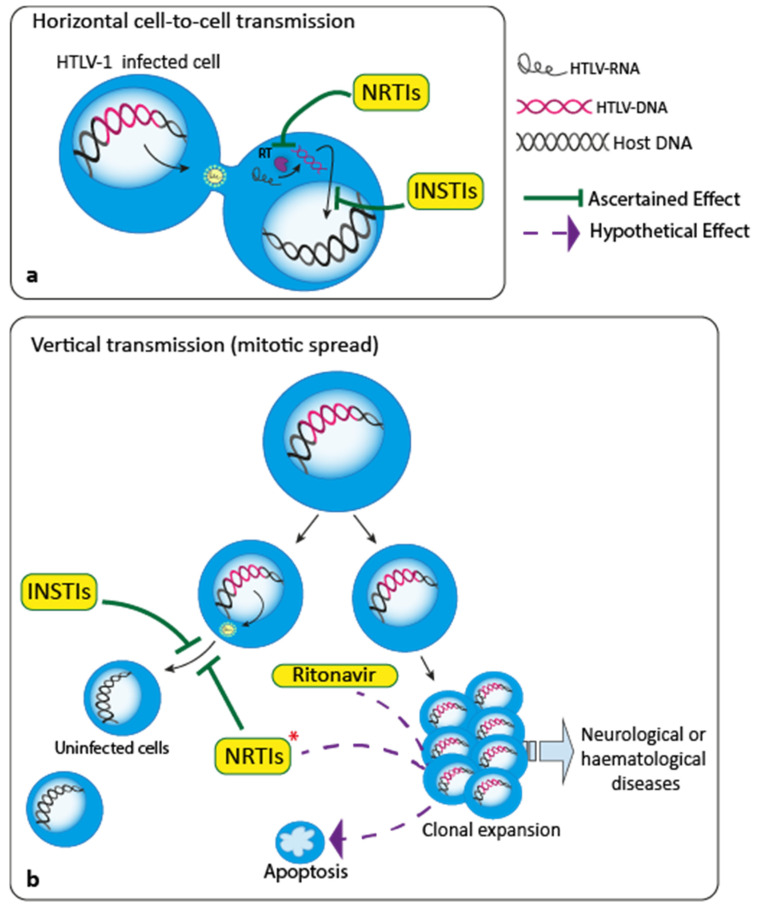
Schematic representation of HTLV-1 transmission and possible mechanisms of antiviral therapy. (**a**) Process by which nucleoside/nucleotide reverse transcriptase inhibitors (NRTIs) and integrase strand transfer inhibitors (INSTIs) interrupt the horizontal cell-to-cell transmission of HTLV-1 from an infected cell to an uninfected cell. (**b**) Processes by which NRTIs, INSTIs and ritonavir could interfere with the vertical transmission of HTLV-1 and consequent insurgence of diseases: this could occur by interrupting the transmission of the virus from infected clones to uninfected cells and by limiting clonal expansion through induction of apoptosis, in combination or not with other compounds. * Note that, among the other NRTIs, AZT has been proved to effectively interrupt the maintaining route of ATL in combination with IFNa, at least transiently.

Collectively, the reported data suggest that antiretroviral therapy in HTLV-1 infection could provide benefit although there is not a common protocol to be suggested. The preclinical studies, have clearly demonstrated that antiretroviral therapies based on drugs translated by the HIV experience efficiently repress HTLV-1 infection, with exception of 3TC owe to a natural resistance of the virus. Nevertheless, the clinical results are still relatively discouraging. Different aspects could account for this discrepancy. The first, as reported, is the scarce reliability of known anti-HIV drugs capable to exert any activity towards the vertical spread of HTLV-1 through clonal expansion of already infected cells. Another related point that might be taken into consideration, based on 3TC experience, is that not all the drugs utilized in HIV infection should exert their optimal activity in HTLV-1 infection. Therefore, the still limited results of antiretrovirals in HTLV-1 might depend, at least in part, on the lack of compounds specifically designed and developed towards HTLV-1. Another aspect that should be taken into consideration is that HTLV-1 preferentially infects through cell-to-cell transmission rather than in a direct virus-to-cell way. Thus, in vitro experimental models might have some limitations owing to this fact. Nevertheless, recent results on new anti-HIV integrase inhibitors and on anti-HIV protease inhibitors in HTLV-1 infection could open new scenarios on usage of antiretrovirals in diseases caused by the virus. Relevant to the later, another aspect to be considered is that antiretroviral compounds could be also endowed with properties displaying activities that exert beneficial effects towards diseases caused by HTLV-1 independently on their antiviral activity and/or in addition to it (Figure 1). Finally, given the fact that early treatment could play a pivotal role in determining the efficacy of an anti-HTLV-1 antiretroviral therapy, the diagnosis of HTLV-1 infection should be improved to establish a preventive program with antiretroviral drugs. In fact, the need to survey HTLV-1 infection more deeply with respect to what has been done until now is also sustained by recent studies showing how HTLV-1 infection could change morbidity and mortality of syndromes other than ATL and or HAM/TSP in endemic areas [57]. Therefore, a better screening and precocious diagnosis of HTLV-1 could encourage the perception that HTLV-1 is not an extremely rare disease [58] and deserves more focused and early treatments.
